# Heterogeneity of microglia and their differential roles in white matter pathology

**DOI:** 10.1111/cns.13266

**Published:** 2019-11-15

**Authors:** Janice Lee, Gen Hamanaka, Eng H. Lo, Ken Arai

**Affiliations:** ^1^ Neuroprotection Research Laboratory Departments of Radiology and Neurology Massachusetts General Hospital and Harvard Medical School Charlestown MA USA

**Keywords:** demyelination, microglia, oligodendrocyte, white matter damage, white matter repair

## Abstract

Microglia are resident immune cells that play multiple roles in central nervous system (CNS) development and disease. Although the classical concept of microglia/macrophage activation is based on a biphasic beneficial‐versus‐deleterious polarization, growing evidence now suggests a much more heterogenous profile of microglial activation that underlie their complex roles in the CNS. To date, the majority of data are focused on microglia in gray matter. However, demyelination is a prominent pathologic finding in a wide range of diseases including multiple sclerosis, Alzheimer's disease, and vascular cognitive impairment and dementia. In this mini‐review, we discuss newly discovered functional subsets of microglia that contribute to white matter response in CNS disease onset and progression. Microglia show different molecular patterns and morphologies depending on disease type and brain region, especially in white matter. Moreover, in later stages of disease, microglia demonstrate unconventional immuno‐regulatory activities such as increased phagocytosis of myelin debris and secretion of trophic factors that stimulate oligodendrocyte lineage cells to facilitate remyelination and disease resolution. Further investigations of these multiple microglia subsets may lead to novel therapeutic approaches to treat white matter pathology in CNS injury and disease.

## 1 INTRODUCTION

White matter primarily comprises myelinated axons that connect neurons in various regions of the brain. Filling in nearly half of the human brain,^1^ growing evidence shows that appropriate myelination in the white matter is required for the development of cognition, memory, motor function, and complex skills.^2^, ^3^, ^4^, ^5^, ^6^ Thus damage or abnormalities in white matter can result in various neuronal diseases, such as multiple sclerosis (MS),^7^, ^8^, ^9^ Alzheimer's disease (AD),^10^, ^11^, ^12^ traumatic brain injury (TBI)^13^, ^14^, ^15^ and vascular cognitive impairment and dementia (VCID) including subcortical ischemic vascular dementia (SIVD).^12^, ^16^, ^17^ Moreover, structural changes in white matter or abnormalities in myelin genes are reported to be the risk factors of psychiatric disorders such as depression,^18^, ^19^, ^20^ schizophrenia,^21^, ^22^, ^23^ and obsessive‐compulsive disorder.^24^, ^25^, ^26^ Therefore, appropriate myelination by oligodendrocytes in the white matter is critical for proper development and maintenance of various brain functions.

Clinical observations of these white matter–related diseases have commonly reported activation of microglia. PET studies in MS and postmortem brain section studies from AD patients showed that the increased population of activated microglial cells correlates with their clinical disability.^27^, ^28^, ^29^, ^30^ Genome‐wide meta‐analysis identified functional pathways influencing AD risk, which included genes related to activation of microglia.^31^, ^32^ Experiments on experimental autoimmune encephalomyelitis (EAE), an animal model of MS, have also revealed correlations between microglial and macrophage activation and the disease as their depletion or inactivation resulted in a delay of the disease onset along with decreased severity of clinical symptoms.^33^, ^34^, ^35^ These observations indicate that the change in microglial activation plays a pivotal role in pathophysiology of various white matter–related diseases.

Microglia are resident immune cells that constitute up to 10 ~ 15% of the cells in CNS. These cells contribute to brain homeostasis by surveying their surrounding microenvironment for indicators of injury or infection known as damage‐associated molecular patterns (DAMPs) or pathogen‐associated molecular patterns (PAMPs).^36^, ^37^, ^38^ Upon encountering these signs microglia quickly become activated to clear cellular debris and dead neurons by phagocytosis. In various neurodegenerative diseases, however, they serve as powerful source of pro‐inflammatory molecules cytotoxic to other components of CNS, including neurons and oligodendrocytes, thus contributing to disease initiation and progression.^39^, ^40^, ^41^, ^42^, ^43^ Interestingly, unlike the conventional detrimental roles of activated microglia, recent studies have also reported their protective effects including regeneration of myelin in the white matter.^44^, ^45^ Therefore, the existence of different microglial subsets and their functional diversity depending on spatiotemporal status of neuronal disorders has risen to be investigated.

In this mini‐review, we survey the diverse microglial changes in white matter during CNS injury and disease, with an emphasis on understanding how these responses affect disease progression and resolution, and the development of potential therapeutic opportunities.

## 2 DIVERSE ACTIVATION OF MICROGLIA IN WHITE MATTER–RELATED DISEASES

As previously mentioned, microglia are found to be “activated” in various white matter–related diseases in a classical way. As competent presenters of antigen, highly activated microglia express molecules for antigen presentation such as major histocompatibility complex II (MHC II) and its costimulatory factors CD40 and CD86 (also known as B7‐2), classical markers of microglia activation, in MS and AD patients.^46^, ^47^, ^48^, ^49^ This was also observed in animal model as microglia in EAE mice underwent proliferation and showed increased expression of CD45, MHCII, CD40, and CD86 as well.^50^ These activated microglia also synthesize a wide range of cytokines, chemokines, cell adhesion glycoproteins, and reactive oxygen radicals, which could be damaging to axons, myelin, and oligodendrocytes.^42^, ^51^, ^52^ Both clinical and animal model studies also revealed that the high proliferation of microglia along with their activation mainly occurred at early stages of the disease particularly at the active sites of demyelination, which was not observed in the later recovery stage.^50^, ^53^, ^54^ These studies demonstrate that classical microglia activation plays critical roles during early stages of MS and AD pathology.

Similar phenomena were observed in ischemic dementia models as well. Inducing white matter lesions in rats by clipping the bilateral carotid arteries to mimic ischemic dementia revealed that microglia and macrophages in white matter showed elevated expression of MHC‐I/II or matrix metalloprotease‐2 (MMP‐2) at 3 days hypoperfusion, indicating their early activation after the onset of cerebral hypoperfusion.^55^, ^56^, ^57^ In mice treated with right unilateral common carotid arteries occlusion (rUCCAO), an animal model for SVID, microglia in the corpus callosum were also found to be activated as the number of ionizing calcium‐binding adaptor molecule 1 (Iba‐1) positive microglia was greatly increased.^58^ Treatment of carnosine (β‐alanyl‐L‐histidine), a natural dipeptide highly expressed in CNS, resulted in microglial deactivation and ameliorated cognitive impairment and white matter lesion in these mice. These results also support that early classical activation of microglia contributes to demyelination at the early stages of white matter diseases.

Interestingly, recent studies have revealed that microglia activation is not a singular phenotypical change but rather a dynamic response that involves spatially and transcriptionally distinct subpopulations. Mrdjen et al performed high‐dimensional single‐cell mapping in EAE mouse CNS and discovered a unique activation profile of microglia.^59^ Although they expressed universal microglia activation markers such as CD44, CD86, and programmed death‐ligand 1 (PDL1), activated microglia from EAE mice showed decreased CD14 expression and increased MHC II and stem cell antigen‐1 (Sca‐1) expressions which were unobserved in those from aged or AD mice, suggesting their unique activation status in EAE. More strikingly, multiple subsets of activated microglia were observed even within the same disease model. Hammond et al carried out single‐cell RNA sequencing of microglia from lysolecithin (LPC) injected mice, which triggers focal demyelination of the subcortical white matter to mimic MS.^60^ This revealed multiple clusters of activated microglia subpopulations with different gene expression profiles in demyelinated lesions. These subsets shared some common gene expressions such as *apoE (apolipoprotein E)* and yet showed unique expressions as well such as *Ccl4* or *Cxcl10* depending on the clusters, indicating that microglia can have multiple forms of activation where both generalized and selective transcriptional programs are delicately orchestrated. These findings were able to be translated to human disease as at least one of the subsets shared the similar gene expression profile to the previously discovered microglia group in human MS lesions,^61^, ^62^ suggesting that these unique markers of activated microglia could potentially be used as biomarkers or therapeutic targets.

Evidence of different activation status of microglia was also observed in ischemic dementia as well depending on the region within the white matter. Simpson et al investigated the microglia of white matter lesions of aged human brains and discovered that periventricular lesions contained significantly more activated microglia as they expressed MHC II, CD40, and B7‐2 than either control white matter or deep subcortical lesions.^63^ Although both microglia found in periventricular and subcortical lesions were activated as they both showed high proliferation rate, the morphological difference existed as microglia in periventricular lesion showed ramified and activated shape, whereas those within subcortical lesions had more ameboid and phagocytic phenotype. This may reflect the different types of “activated” microglia subsets depending on the lesion site as previously observed in the mouse MS model.^60^

Moreover, recent studies have revealed that microglial subtype can be altered depending on the disease pathology. Locatelli et al utilized a real‐time in vivo imaging in EAE mice and observed the evolution of microglia and mononuclear macrophages in a spatiotemporal manner.^64^ Interestingly, their molecular expression patterns were switched from pro‐inflammatory markers such as inducible nitric oxide synthase (iNOS) to immuno‐regulatory ones including arginase1, suggesting that their subtypes have changed. The highest rates of these conversions were observed in initial lesions and increased over time, peaking during lesion resolution. This finding demonstrates that microglial subtype can be regulated depending on spatial and temporal status of EAE and this transition may affect disease progression and resolution. Another study done in TBI model reported a transition in microglial subtype as well. When TBI was induced in mice by a controlled cortical impact (CCI), microglia and macrophages expressed early immuno‐regulatory phenotype after the impact but was gradually replaced by the pro‐inflammatory phenotype at the site of injury.^13^ Notably, the severity of white matter injury was strongly correlated with the number of pro‐inflammatory microglia and macrophages, suggesting that this subset could be a possible therapeutic target.

Collectively, these findings suggest that multiple subpopulations of activated microglia exist depending on the disease type or spatiotemporal status of the disease and they contribute to progression and resolution of various white matter–related diseases.

## 3 MICROGLIAL ACTIVITY IN WHITE VS GRAY MATTER

After white matter damage, clearance of myelin debris from demyelination is critical for oligodendrocyte precursor cell (OPC) recruitment and their maturation into oligodendrocytes.^65^, ^66^, ^67^ Therefore, the phagocytic activity of microglia is important not only for regional clearance but also for efficient remyelination.^67^, ^68^, ^69^ Could this phagocytic activity of microglia be also differentially regulated like its activation status? In normal human brain, white matter contained significantly higher number of microglia and macrophage than gray matter,^70^ which was also observed in adult rat brain as well.^71^ Moreover, these microglia and macrophages in the normal human white matter expressed more phagocytosis‐related proteins such as CD68 and CD86 compared to the gray matter,^72^ indicating that the basal level of microglial phagocytosis is higher in the white matter. After ischemic damage, the microglia within the lesion were all highly activated in both white and gray matter, but during the late scaring phase less population of white matter microglia expressed purinergic receptor P2Y12 (P2RY12), a “resting” marker than the gray matter, suggesting a differential regulation of microglial activity depending on the region of the brain.

Similar results were observed in animal models as well. In TgAPP21 transgenic rats, an AD model, microglial activation and accumulation was significantly stronger in white matter tract including supraventricular corpus callosum (SVCC) than gray matter.^73^ Notably, this SVCC microglial activation was found to be positively correlated to impaired executive functioning, suggesting that this may contribute to the underlying mechanism of the impairment. Moreover, when mice were fed with cuprizone to induce demyelination especially in the corpus callosum area, microglial cell response was found to be stronger in the white matter than in gray matter as the accumulation of CD107b (Mac3) positive‐activated microglia was significantly higher in corpus callosum than the cortex.^74^ Interestingly, the cortex layer closer to the corpus callosum contained more activated microglia, suggesting that the high content of myelinated fibers or proximity to white matter may affect the activation and proliferation of microglia.

To investigate how white and gray matter may contribute to this difference, Poel et al performed a transcriptional profiling of microglia from white and gray matter using postmortem tissues of both control and MS donors.^75^ In both groups, a clear region‐specific profile of microglia was observed, as microglia in gray matter showed higher expression of type‐1 interferon genes, whereas those in white matter showed higher expression of NF‐kB pathway‐related genes. In MS lesions, the increased expression of lipid metabolism‐related genes was observed in white matter microglia, while in gray matter the expression of glycolysis and iron homeostasis‐associated genes were found to be elevated. These differential gene profiles support the difference in microglial subsets between the white and gray matter.

The differential profile of microglia may be due to its surrounding environment. Huizinga et al conditioned mouse macrophage cell line J774.2 by incubating with either gray or white matter from humans and measured their phagocytic activity and found that the number of cells containing myelin or neuronal antigens was significantly higher in cells incubated with white matter.^76^ Although there may be different mechanisms involved between macrophages and microglia, the finding suggests the existence of white matter‐specific factors that can boost the phagocytic activity. One of the factors could be the OPCs in the white matter, as Liu et al recently discovered that selective OPC depletion using small‐molecule inhibitors of platelet‐derived growth factor (PDGF) signaling abolished the homeostatic microglia signature but did not change their disease‐associated profiles in cultured brain slices.^77^ Similar findings were also observed in *vivo* by inducing conditional depletion of OPCs in adult mouse brain, suggesting that OPC has a crucial influence onto cellular states of microglia which are relevant to neurodegenerative diseases. Although a small population of OPCs exists in the gray matter as well,^78^ they are reported to be mostly quiescent or slowly proliferating,^79^ whereas OPCs in white matter continuously generate mature and myelinating oligodendrocytes in response to PDGF signaling,^79^, ^80^ showing that OPCs have different characteristics depending on its region. These all suggest that white matter OPCs may exhibit specific effects on white matter residing microglia, differentiating them from gray matter residing ones.

Overall, the above findings demonstrate that activities of microglia in white matter could be differentially regulated from gray matter, hence, may result in different outcome in terms of remyelination and disease resolution (Table 1).

**Table 1:** Comparison of microglial traits between white and gray matter

CNS status	Traits	White matter	Gray matter	Reference
Basal or normal	Microglial cell number	Higher	Lower	70, 71
	Expression of phagocytic markers	Higher	Lower	72
	Expression of type‐I interferon genes	Lower	Higher	75
	Expression of NF‐κB pathway genes	Higher	Lower	75
Disease or disease model	Expression of resting markers after ischemic damage	Lower	Higher	72
	Accumulation of activated microglia in AD mouse model	Dramatic increase	Mild increase	73
	Accumulation of activated microglia after demyelination	Dramatic increase	Mild increase	74
	Expression of lipid metabolism genes in MS	Elevated	No significant changes	75
	Expression of genes associated with glycolysis and iron homeostasis in MS	No significant changes	Elevated	75
	Phagocytic activity when treated to macrophage cell line J774.2	Dramatic increase	Mild increase	76

Abbreviations: AD, Alzheimer's disease; CNS, central nervous system; MS, multiple sclerosis; NF‐kB, nuclear factor kappa‐light‐chain‐enhancer of activated B cells.

## 4 MICROGLIA‐DERIVED FACTORS AND OLIGODENDROCYTES LINEAGE CELLS

Aside from its phagocytic activities, multiple studies have shown that activated microglia secrete various factors that directly affect the fate of OPCs and oligodendrocytes, key component cells of white matter homeostasis and repair. Traditionally, activated microglia were considered to secrete pro‐inflammatory factors involved in the initiation and propagation of inflammatory cascade thus promoting demyelination in neural disorders. LPS‐activated microglia, polarized to pro‐inflammatory status, secreted tumor necrosis factor alpha (TNFα) and interleukin‐1beta (IL‐1β), both known to be cytotoxic to oligodendrocytes.^81^, ^82^, ^83^ Indeed, in rat primary oligodendrocyte cultures, co‐treatment of TNFα and cuprizone resulted in a significant decrease in their cell viability.^84^ When microglial activation was blocked by treatment of minocycline in cuprizone administered mice, the demyelination was prevented suggesting the detrimental roles of microglia‐derived factors on oligodendrocytes. In addition, a positive correlation between nitric oxide (NO_2_) production by microglia and death of oligodendrocytes was observed in rat cells, suggesting NO_2_ ‐induced damage as the cytotoxic mechanism for oligodendrocytes.^85^ In another study, LPS‐activated microglia secreted heat shock protein 60 (HSP60), a stress chaperone protein that has dual functions in cell apoptosis depending on specific conditions. These secreted HSP60 were found to bind to Toll‐like receptor (TLR) 4 of OPCs and initiated its apoptotic mechanism.^86^

On the other hand, recent studies have revealed protective roles of microglia during the remyelination phase. After myelin damage in MS model, a genome‐wide gene expression analysis was performed for the microglia in corpus callosum during remyelination. A repertoire of cytokines and chemokines such as *Cxcl10, Tgfb1, Pdgfa,* and *Pdgfb* were found to be expressed which are known to recruit OPCs to the lesion site and promote their differentiation, thus possibly facilitating the remyelination process.^44^ Transforming growth factor α (TGFα) was also found to be secreted by microglia after ischemic damage and showed protective roles on OPCs and oligodendrocytes in vitro and contributed to white matter integrity in vivo.^87^ Moreover, multiple factors including activin A, galectin‐3, and insulin‐like growth factor 1 (IGF1) were produced by microglia which supported oligodendrocyte differentiation in vivo.^88^, ^89^, ^90^ Interestingly, TNFα and IL‐1β, mentioned earlier as oligodendrocyte‐damaging factors secreted by microglia, were found to play beneficial roles in remyelination as well along with other pro‐inflammatory factors such as CXCL13 and endothelin‐2.^91^, ^92^, ^93^ These contrasting effects were driven by time‐dependent expression change of their different receptor subtypes or their promotion of secondary proremyelinating factor secretion in the later phase of disease onset, demonstrating complex roles of microglia in regulating the fate of oligodendrocyte lineage cells. Thus, delicate orchestration of these microglia‐derived factors could play critical roles in white matter disease onset and resolution.

This regulation of secretion factors could be influenced by different activation states of microglia. Miron et al discovered that the microglial and macrophage switch from pro‐inflammatory to immuno‐regulatory occurred over time after inducing focal demyelination by LPC in the mouse corpus callosum. These regulatory populations secreted higher levels of IGF1 and activin A than those of pro‐inflammatory phenotypes and promoted oligodendrocyte differentiation.^45^ Yu et al^94^ also reported similar findings as when microglia was polarized to regulatory state by inducing homeobox gene msh‐like homeobox‐3 (*Msx3*), levels of activin A, and IGF1 mRNAs were increased and facilitated remyelination in mice MS models. Moreover, Wang et al discovered that treatment of histone deacetylase (HDAC) inhibitor scriptaid also triggered the beneficial switch as it resulted in less production of TNFα and NO by microglia and macrophages with increased preservation of co‐cultured oligodendrocytes in vitro and prevented white matter damage in vivo.^95^ Notably, some microglial subset can be induced by neighboring microglia. When signal regulatory protein α (SIRPα), a membrane protein, was genetically ablated, the number of CD11c + microglia was significantly increased in white matter of the brain.^96^ Microglia‐specific ablation of SIRPα alleviated cuprizone‐induced demyelination, suggesting that microglial SIRPα suppresses the induction of CD11c + microglia and demyelination damage in white matter, possibly involving indirect roles on oligodendrocyte lineage cells. These studies indicate that depending on the subset of microglia the expression of derived factors can be altered, thus causing contrasting effects on oligodendrocytes.

**Figure 1:**
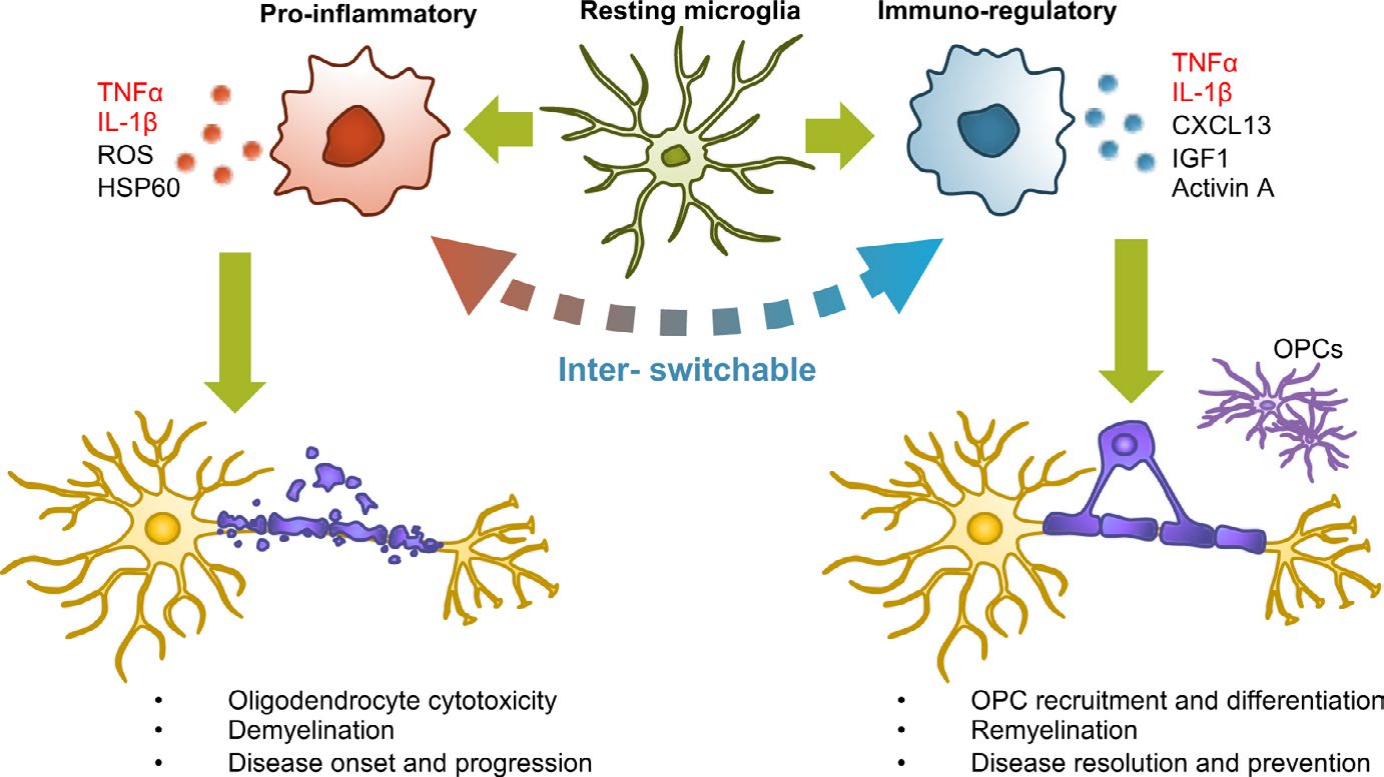
Effects of microglia‐derived factors on oligodendrocyte lineage depending on microglial subsets. A pro‐inflammatory microglial subset secretes cytokines and reactive oxygen species (ROS) that can directly damage oligodendrocytes thus resulting in demyelination. Immuno‐regulatory subsets secrete trophic factors that promote OPC migration and differentiation thus resulting in remyelination. Switching the pro‐inflammatory subset into an immuno‐regulatory subset may represent a potential therapeutic approach for rescuing white matter in CNS injury and disease. Note that microglia‐derived TNFα and IL‐1β can both be cytotoxic or beneficial to oligodendrocyte lineage cells (marked in red). These complex effects are likely to be dependent on signals from neighboring cells and surrounding environmental conditions. Future studies are warranted to investigate how and why identical factors from microglia can show opposite effects on oligodendrocyte damage/recovery

These findings above demonstrate the untraditional subset of microglia that secretes trophic factors for OPC generation, migration and maturation, revealing the complex nature of microglia population. Altogether, depending on the pathological stage of white matter damage, different subsets of microglia could affect the fate of OPCs and oligodendrocytes by secreting factors of various effects (Figure 1).

## 5 POTENTIAL THERAPEUTIC OPPORTUNITIES

Currently, no therapies designed to improve remyelination are approved, especially none targeted directly to microglia.^97^ However, several drugs undergoing clinical trials to directly enhance OPC differentiation have shown off‐target effects that reduced neuroinflammatory activity of microglia,^98^ raising the possibility that microglia could be directly targeted as well. In this section, we introduce recent studies that reported potential therapeutics associated with modulation of microglia phenotype that can improve white matter integrity in various injury models.

In mice induced with ischemic stroke model, hypothermia decreased the inflammatory phenotype and increased the immuno‐regulatory type of microglia along with decrease in their total number compared to the control group, which promoted the long‐term integrity of the white matter.^99^ Similarly, treatment of VK‐28 (5‐[4‐(2‐hydroxyethyl) piperazine‐1‐ylmethyl]‐quinoline‐8‐ol), a brain‐permeable iron chelator to intracerebral hemorrhage model of mice, polarized microglia to immuno‐regulatory subtype and deceased white matter injury.^100^ Treatment of dimethyl fumarate, a drug known to suppress microglial inflammation, to mice with severe brain hypoperfusion resulted in modest decrease in the number of inflammatory microglia and macrophages and improved functional impairment in the white matter.^101^ Moreover, in rat TBI model, rats co‐treated with minocycline and N‐acetylcysteine showed altered ratio of pro‐inflammatory vs immuno‐regulatory population of microglia along with increased remyelination and improved cognition and memory.^14^ Overall, these studies support the idea that directly targeting and converting microglial subtypes could be a novel and efficient therapy for various white matter diseases.

For the microglial phenotype switching strategy to be developed successfully, fully understanding the difference between various microglial subtypes will be beneficial. For instance, if unique biomarkers of specific subset of microglia in certain disease could be validated, specifically ameliorating this subset and maintaining other beneficial population could facilitate the resolution of the disease. As recent single‐cell sequencing studies have already discovered genes that can potentially be used as biomarkers such as *Ccl4* or *Cxcl10* in MS‐related microglia,^60^ targeted drug designing could prove to be an efficient strategy minimizing the side effects. Moreover, if the molecular difference between the demyelination and remyelination promoting microglial subsets could fully be understood, finding a way to switch to the beneficial population will also be a novel therapeutic approach. As overexpressing MSX3 polarized microglia into regulatory state and promoted remyelination in mice,^94^ discovering and validating these “switching factors” is expected to be a first step to develop the microglial phenotype switchig strategy for white matter‐related diseases.

Another potential strategy is the targeted drug delivery to specific brain regions. As activated microglial subpopulation is also diverse depending on their location within the brain, bioengineering of lesion‐specific drug delivery platforms should be considered as well. As several studies have reported that white matter residing microglia tend to have different activation status compared to those of the gray matter, specific delivery of microglia‐targeted drug to white matter could prove to be beneficial to the disease resolution. Moreover, as studies have revealed not only inter‐regional but also intra‐regional differences exist between the microglial subsets, uncovering the region‐specific markers and mechanisms and region‐specific drug delivery could prove to be a novel and efficient strategy. Of course, there is always a risk that phenomena observed in experimental models can often be different from actual human disease pathology. Therefore, the thorough identification of microglia subtypes in cell or animal experimental models must be carefully investigated with clinical samples first to confirm whether they could actually be translated in human disease in order to develop these strategies.

## 6 CONCLUSIONS AND FUTURE OPPORTUNITIES

Microglia were traditionally considered as deleterious cells in CNS disease. In white matter, excessive microglial activation damages neuronal axons and myelin sheaths. However, an emerging literature now suggests a more nuanced scenario with both damaging and beneficial microglia phenotypes. As discussed in this mini‐review, recent advances in molecular technologies now allow one to detect multiple subsets of microglia within the broadly defined “activated” states. After white matter damage, microglia are activated in multiple forms depending on various conditions such as their proximity to the lesion, location within the brain, and the type of disease. During the acute stage, deleterious microglia promote an inflammatory microenvironment involving antigen presentation and secretion of pro‐inflammatory molecules that trigger demyelination and neuronal damage. During the later recovery period, however, excessive microglial activation is toned down and their phagocytic activity contributes to clearance of myelin debris and dead cells. Immuno‐regulatory microglia also secrete factors that promote OPC migration and their maturation into oligodendrocytes, thus facilitating the remyelination process. Although accumulating evidence of these multiple damaging vs beneficial roles has been reported,^102^, ^103^ the mechanisms involved in these contrasting phenomena remain to be fully understood. How can one quantitatively distinguish and map the various microglia phenotypes, and is it possible to manipulate these microglia subsets for therapeutic benefit? After CNS injury and disease, heterogenous populations of activated microglia significantly influence white matter response. Understanding the molecular regulation and functional properties of microglia subsets should contribute to the development of novel therapies for white matter treatments in CNS injury and disease.

## CONFLICTS OF INTEREST

The authors declare no conflict of interest.
